# Brain potentials predict substance abuse treatment completion in a prison sample

**DOI:** 10.1002/brb3.501

**Published:** 2016-05-31

**Authors:** Brandi C. Fink, Vaughn R. Steele, Michael J. Maurer, Samantha J. Fede, Vince D. Calhoun, Kent A. Kiehl

**Affiliations:** ^1^ Department of Psychiatry and Behavioral SciencesClinical and Translational Science CenterThe University of New MexicoAlbuquerqueNew Mexico; ^2^ Intramural Research ProgramNeuroimaging Research BranchNational Institute of Drug AbuseNational Institutes of HealthBaltimoreMaryland; ^3^ The Mind Research Network and Lovelace Biomedical and Environmental Research InstituteAlbuquerqueNew Mexico; ^4^ Department of PsychologyThe University of New MexicoAlbuquerqueNew Mexico; ^5^ Department of Electrical and Computer EngineeringThe University of New MexicoAlbuquerqueNew Mexico

**Keywords:** Event‐related potentials, pattern classifier, principal component analysis, prison inmate, substance abuse treatment, support vector machine

## Abstract

**Introduction:**

National estimates suggest that up to 80% of prison inmates meet diagnostic criteria for a substance use disorder. Because more substance abuse treatment while incarcerated is associated with better post‐release outcomes, including a reduced risk of accidental overdose death, the stakes are high in developing novel predictors of substance abuse treatment completion in inmate populations.

**Methods:**

Using electroencephalography (EEG), this study investigated stimulus‐locked ERP components elicited by distractor stimuli in three tasks (VO‐Distinct, VO‐Repeated, Go/NoGo) as a predictor of treatment discontinuation in a sample of male and female prison inmates. We predicted that those who discontinued treatment early would exhibit a less positive P3a amplitude elicited by distractor stimuli.

**Results:**

Our predictions regarding ERP components were partially supported. Those who discontinued treatment early exhibited a less positive P3a amplitude and a less positive PC4 in the VO‐D task. In the VO‐R task, however, those who discontinued treatment early exhibited a more negative N200 amplitude rather than the hypothesized less positive P3a amplitude. The discontinuation group also displayed less positive PC4 amplitude. Surprisingly, there were no time‐domain or principle component differences among the groups in the Go/NoGo task. Support Vector Machine (SVM) models of the three tasks accurately classified individuals who discontinued treatment with the best model accurately classifying 75% of inmates. PCA techniques were more sensitive in differentiating groups than the classic time‐domain windowed approach.

**Conclusions:**

Our pattern of findings are consistent with the context‐updating theory of P300 and may help identify subtypes of ultrahigh‐risk substance abusers who need specialized treatment programs.

## Introduction

Studies have estimated that 50–80% of prison inmates meet diagnostic criteria for a substance use disorder (SUD), and up to 49% participate in some form of substance abuse treatment during incarceration (Mumola and Karberg 2004). The successful treatment of SUDs in prison inmates is a significant public health concern. The period shortly after release from custody represents a significant risk for the return to substance use and accidental overdose deaths. For example, 76% of deaths of former prison inmates within the 2 weeks of release and 59% of deaths within 3 months of release are attributable to drug‐related causes (Merrall et al. 2010). Because the stakes are high with inmate populations and more treatment is consistently associated with reduced substance use (Simpson et al. 1997; Mattson et al. 1998; Gossop et al. 2002; Sayre et al. 2002; Hubbard et al. 2003), the impetus to discover novel predictors of treatment discontinuation is significant with an inmate population.

The functional integrity of the central nervous system in substance‐abusing and substance‐dependent individuals has been widely investigated using quantitative electroencephalography (QEEG) and event‐related potentials (ERPs). QEEG studies have been able to distinguish between healthy control groups and abusers of cocaine (Bauer 2001b), methamphetamine (Kalechstein et al. 2009), and between ecstasy users with high and low cumulative ecstasy use (Adamaszek et al. 2010). Also, an investigation of cortical complexity in methamphetamine‐dependent individuals using electroencephalography (EEG) not only indicated less complexity but also correlated with other clinical features such as patterns of use (Yun et al. 2012). Similarly, ERP studies, particularly those investigating the P300, have found that substance‐dependent users of cocaine, cocaine and alcohol, and opioids (Bauer 2001a), evidence reduced P300 amplitude compared to their healthy controls.

In addition to being able to distinguish substance abusing groups from healthy controls, previous studies have investigated N200 and P300 ERP components in relation to treatment outcomes for a variety of disorders. A large literature has emerged that focuses on N200 in the process of cognitive control which includes the monitoring and processing of feedback that is used in strategy updating which includes response inhibition, response conflict, and error monitoring. In a sample of children in treatment for externalizing behavior problems, Woltering et al. (2011) found that larger N200 amplitudes and smaller frontal P300 amplitudes characterized the clinical children, and reflected less efficient response inhibition (N200) and less efficient processing of context updating or response control (P300). After treatment, treatment effects were specific to N200. Children who improved after treatment showed marked reductions in N200, which the authors discussed as reflecting fewer cortical resources being devoted to response inhibition. In studies of substance abuse treatment completion with community samples, reduced P300 amplitude has been associated with treatment discontinuation and relapse (Bauer 1997, 2001a; Anderson et al. 2011). Reduced P300 amplitudes associated with treatment discontinuation and relapse are typically elicited during processing of task‐irrelevant distractor stimuli. The distractor P300 has an anterior topography (i.e., P3a) and is typically interpreted as reflecting inhibitory and orienting processes (see Polich 2007 for a review). In addition, a recent investigation by our group found that multiple ERP components (less positive stimulus‐locked P2, less negative response‐locked ERN/Ne, and increased response‐locked Pe amplitudes) indexing perceptual gating and error processing were associated with early substance abuse treatment discontinuation in a sample of inmates with SUDs (Steele et al. 2014). As we discussed in this study, less positive stimulus‐locked P2 is thought to index early sensory gating and the ability of individuals to filter extraneous information when allocating attention (Boutros et al. 2006; Lijffijt et al. 2009), and Pe is thought to index conscious evaluation of errors, response strategy adjustments, and/or affective assessment of error (Nieuwenhuis et al. 2001; Overbeek et al. 2005). We felt that our findings may reflect deficiencies in neural correlates of early sensory gating which may be linked to reduced working memory stores and deficiencies in subsequent response inhibition, as well as deficiencies in conscious evaluation of errors and response strategy adjustments. We felt that it was possible that these deficiencies may be related to difficulty processing information in treatment, evaluating the consequences of their substance use, and the benefits of treatment which may contribute to early treatment discontinuation. Findings such as these provide compelling evidence for further investigation of neurocognitive information processing correlates in incarcerated substance using individuals.

Processing of distractor stimuli is believed to engage a number of cognitive processes, including response inhibition, novelty detection, and orienting processes. Based on previous findings of reduced P300 amplitude in substance abusing individuals (Bauer 1997; Bauer and Hesselbrock 1999) that occurred in response to low probability, repeated, nontarget stimuli, or *distractors,* we investigated stimulus‐locked ERP components elicited by distractor stimuli in three different paradigms to isolate various P3a waveforms to examine whether they were useful predictors of treatment completion. The three tasks used in this study were designed to provide further refinement of the neurocognitive measures implicated in treatment discontinuation, and to identify how well novelty processing (with and without stimulus rarity) and response inhibition predicted substance abuse treatment completion outcomes. We hypothesized that those who discontinued SUD treatment early, compared to those who completed treatment, would exhibit a less positive P3a amplitude elicited by distractor stimuli. The less positive or abnormal P3a reflects attentional impairments in processing of novel (or distractor) stimuli, and we expected attentional impairments to be directly related to stimulus novelty tested in the three tasks of this study. More specifically, we hypothesized that the P3a response to a task where the novel visual stimuli are distinct (visual oddball – distinct; VO‐D) would be more predictive of treatment discontinuation than the P3a response to a task where the distractor stimuli in a visual oddball task are repeated (visual oddball – repeated; VO‐R). Furthermore, we hypothesized that the P3a responses to these oddball tasks would be more predictive than those obtained in a Go/NoGo task developed to elicit a P3a response in the absence of any novelty response or probability manipulations and where the distractor stimuli are presented with equal probability (Go/NoGo).

## Materials and Methods

### Sample and participant selection

Participants included in this study were 123 (77 females) treatment‐seeking incarcerated individuals who were recruited from two medium‐security prisons in the state of New Mexico, and were part of a larger study investigating neurocognitive changes associated with a novel behavioral treatment for drug abuse of cocaine, methamphetamine, or heroin (NIDA R01 DA020870). Participants included in this study identified their drug of choice as cocaine (*N* = 53; 12 discontinued), methamphetamine (*N* = 51; 7 discontinued), or heroin (*N* = 19; 6 discontinued). The mean age of participants was 34.70 years (SD = 8.74) at the time of the baseline assessment when EEG was collected, and when the participants were randomized into one of three 12‐week manualized psychological interventions. Because each treatment type was well represented (Addictions Counseling [AC], *N* = 39; Relapse Prevention [RP; Marlatt and Gordon 1985], *N* = 49; Substance Expectation Therapy [SET; Jaffe and Wilber 2001], *N* = 34; one participant discontinued treatment before treatment group assignment) and the completion proportion of each group (completion group: AC, *N* = 32; RP, *N* = 37; SET, *N* = 29; discontinuation group: AC, *N* = 7; RP, *N* = 12; SET, *N* = 5; unassigned, *N* = 1) were similar, we collapsed across treatment types to yield maximum power. Treatment sessions occurred once weekly, and it should be noted that most participants were treatment naïve and none participated in any other substance abuse treatment, including mutual‐help groups, while participating in our study. Sixty‐seven percent of the sample self‐identified as Hispanic, 26% White, 3.2% Native American/American Indian, 3% Black/African American, and 0.8% selected Other. Approximately 12% of the entire sample was left‐hand dominant; 8.5% of the VOR sample, 9.4% of the VOD sample, and 7.6% of the Go/NoGo task identified themselves as left‐hand dominant.

Ninety‐eight (63 females) participants completed the therapy protocol (i.e., at least nine sessions of the 12‐session protocol (Jaffe and Wilber 2001), and 25 (14 females) participants discontinued treatment before completing the therapy protocol (i.e., receiving eight or fewer sessions). Individuals who did not complete 9 weeks of treatment for reasons other than voluntarily discontinuation (e.g., early release from prison or paroled, *N* = 1, transferred to another facility *N* = 2, or enrolled in another drug treatment program *N* = 2) were not included in the analyses. Of the participants completing informed consent and screening for the study, 123 (46 males, 77 females) were assigned to one of the three therapy cells using a pseudorandomization process. Of the 123 participants included in this study, 94 (57 females) participants completed the visual distractor (VO‐R) task, 96 (56 females) participants completed the visual oddball (VO‐D) task, and 66 (35 females) participants completed the Go/NoGo task, and were included in the present analysis. The same order of task administration was used for each participant: VO‐R; VO‐D; Go/NoGo. Some participants decided not to finish all three tasks which contributed to the unequal sample numbers across tasks.

#### Inclusion criteria

Participants included in this study met the following inclusion criteria: (1) currently incarcerated, (2) cocaine, methamphetamine, or heroin dependent at time of incarceration, (3) no history of head injury resulting in significant loss of consciousness, (4) no history of psychosis or first‐degree relative with psychosis, (5) a sixth‐grade English reading level, and (6) an estimated IQ greater than 70.

### Procedures and ethical considerations

Initial contact was made with potential study participants through announcements by research staff at the correctional facilities. Meetings were scheduled with interested participants where screening was conducted, and informed consent was obtained. Participants were informed of their right to discontinue participation at any point and that their participation was in no way associated with their status at the facility, their parole status, and that there were no direct institutional benefits. Participants received remuneration at the rate of the hourly wage at the facility. All procedures were approved by the Human Research Review Committee at the research institution and correctional facilities where the study was conducted.

### Assessment measures

Trained researchers administered several instruments. There were no significant differences between the treatment completion and treatment discontinuation groups on these measures, *t*'s <1.5 (Table 1; see Tables S1–S3 for task‐by‐group comparisons).

**Table 1:** Descriptive statistics and independent samples *t*‐tests for variables used as covariates – total sample

Variable	All participants (*n *=* *123)			Completed group (*n *=* *98)			Discontinued group (*n *=* *25)			*t*	df	*P*
	*n*	Mean	SD	*n*	Mean	SD	*n*	Mean	SD			
Age	123	34.70	8.74	98	34.83	8.76	25	34.20	8.80	−0.32	121	0.75
IQ	122	95.84	10.32	97	96.43	10.55	25	93.52	9.20	−1.26	120	0.21
Months of abuse	111	535.08	297.74	89	521.18	280.37	22	591.32	361.71	0.99	109	0.33
PCL‐R total	102	20.48	6.10	85	20.62	6.24	17	19.78	5.45	−0.52	100	0.61
PCL‐R‐F1	98	5.42	2.88	81	5.62	3.01	17	4.47	1.97	−1.50	96	0.14
PCL‐R‐F2	101	13.06	3.36	84	13.07	3.26	17	13.00	3.92	−0.08	99	0.94
Precontemplation	112	54.51	10.38	90	54.61	10.47	22	54.09	10.19	−0.21	110	0.83
Contemplation	111	41.76	13.76	90	41.61	13.57	21	42.38	14.88	0.23	109	0.82
Action	113	48.76	12.69	91	48.68	13.08	22	49.09	11.20	0.14	111	0.41
Maintenance	112	46.61	10.12	91	46.48	9.56	21	47.14	12.51	0.27	110	0.79
State anxiety	100	39.87	12.66	81	39.63	12.57	19	40.89	13.33	0.39	98	0.70
Trait anxiety	98	43.72	10.00	79	43.70	10.33	19	43.84	8.75	0.06	96	0.96
Depression	121	15.72	11.15	98	15.45	11.38	23	16.87	10.26	0.55	119	0.58

All participants (*n *=* *123) either successfully completed or discontinued a cognitive behavioral substance abuse treatment program. Individuals in the completed group (*n *=* *98) include adult incarcerated offenders who successfully completed 9 weeks of a cognitive behavioral substance abuse treatment program. Individuals in the discontinued group (*n *=* *25) include adult incarcerated offenders who discontinued treatment prior to 9 weeks of a cognitive behavioral substance abuse treatment program. Assessments: Intelligence quotient (IQ) was calculated from the Wechsler Adult Intelligence Scale III (WAIS‐III; Wechsler 1997); Months of Abuse is the total number of months of abuse calculated by a modification of the Addiction Severity Index (ASI‐X; McLellan et al. 1992); PCL‐R‐F1 and PCL‐R‐F2 are the Factor 1 and Factor 2 summary scores from the Psychopathy Checklist – Revised (PCL‐R; Hare 2003); Precontemplation, Contemplation, Action, and Maintenance are summary scores of subscales from the University of Rhode Island Change Assessment (URICA; McConaughy et al. 1983); State Anxiety and Trait Anxiety are summary scores from the State and Trait Anxiety Questions from the State‐Trait Anxiety Inventory (STAI; Spielberger et al. 1983); Depression is the total score from Beck Depression Inventory‐II (BDI‐II; Beck et al. 1996).

#### Depressive symptoms

Depressive symptoms were assessed using the Beck Depression Inventory‐II (BDI‐II, (Beck et al. 1996), a 21‐item measure that assesses the severity of depressive symptoms. Depressive symptom measures were unavailable for nine participants. The Cronbach's alpha for the BDI‐II in this sample was 0.74.

#### Anxiety symptoms

Anxiety symptoms were assessed using the State‐Trait Anxiety Inventory (STAI; (Spielberger et al. 1983), a 40‐item measure that assesses the intensity of anxiety symptoms and distinguishes between state anxiety and trait anxiety. Anxiety symptoms were unavailable for nine participants. The Cronbach's alpha for the STAI in this sample was 0.94.

#### Psychopathy

Psychopathy was assessed using the PCL‐R (Hare 2003) comprising two factors; Factor 1 assessing callous interpersonal and affective traits, and Factor 2 assessing impulsive lifestyle and antisocial behavior. In this study, Factor 2 was used as an index of impulsivity. Factor 1 scores were unavailable for 15 participants and Factor 2 scores were unavailable for 19 participants. The Cronbach's alpha for the PCL‐R in this sample was 0.85.

#### Motivation for change

Motivation for change was assessed using the University of Rhode Island Change Assessment (URICA; McConaughy et al. 1983), a continuous measure of four stages of how a person may feel about making changes before they acknowledge they have a problem (precontemplation), after they acknowledge they have a problem (contemplation), once they take steps toward treatment (action), and maintaining the change (maintenance). URICA scores were unavailable for eight participants. The Cronbach's alpha for the URICA in this sample was 0.71.

#### Estimated IQ

IQ was estimated for the sample using the Vocabulary and Matrix Reasoning subtests of the Wechsler Adult Intelligence Scale (Wechsler 1997; *M *=* *95.84, SD = 10.32).

#### Reading level

Reading level was assessed using the Reading Subtest from the Wide Range Achievement Test – 3 (Wilkinson 1993).

#### Drug dependence

Drug dependence at the time of incarceration was assessed using the Substance Use Disorder Module from the Structured Clinical Interview for the DSM‐IV, Non‐patient Version (SCID‐I‐NP; First et al. 2002).

### Experimental tasks

Participants performed tasks which differed only on type of stimuli. In each task, two runs were completed; 240 trials in run 1 and 243 trials in run 2. The trial order and counts were optimized for functional magnetic resonance imaging data collection and interpretation. As mentioned previously, the EEG data discussed here were collected as part of a larger study where fMRI was also collected. Therefore, experimental methods were harmonized between the modalities to enhance comparisons which precluded counterbalancing the presentation of tasks to participants during EEG data collection. Each of the tasks consisted of the three stimulus types including frequent stimuli which occurred at 0.80 probability and two infrequent stimuli (target and novel/distractor) that each occurred at 0.10 probability. Each stimulus was presented for 230 ms. Infrequent stimuli were always preceded by at least three frequent stimuli (range three to five). The intervals between stimuli of interest (i.e., infrequent stimuli) were allocated in a pseudorandom manner in a range 8–12 sec. Participants were instructed to respond as quickly and as accurately as possible with their right index finger using a response button box every time a target stimulus was presented. Prior to beginning the task, each participant performed a practice block of 10 trials to ensure an understanding of the instructions.

#### Visual distractor (VO‐R) task

This task consisted of three types of visual stimuli; frequent stimulus (alphabet “T” white text in a black background), infrequent target stimulus (alphabet “X” white text in a black background), and infrequent distractor stimuli (alphabet “C” white text in a black background; Bauer 2001a).

#### Visual oddball (VO‐D) task

This task consisted of three types of visual stimuli; frequent stimulus (a 6 × 6 cm white square on a black background), infrequent target stimulus (a 4 × 4 cm white square on a black background), and infrequent novel stimuli (colored nonrepeating shapes on a white box in a black background; Kiehl et al. 1999). Each novel stimulus was a different combination of geometry and color such that no repetition occurred throughout the task. The larger nontarget stimuli subtended a visual angle of 8.5 by 8.5 degrees, and the smaller target stimuli subtended a visual angle of 3.8 by 3.8 degrees.

#### Equal probability Go/NoGo task

This task consisted of three types of visual stimuli; frequent stimulus (a black screen), infrequent target stimuli (a white “X” on black background), and infrequent nontarget “NoGo” stimuli (a white “K” on black background; Kiehl et al. 1999).

### Electroencephalographic recordings

EEG data were collected in a small room in an area separate from the general population housing provided by the facility. After placement of the electrodes, participants were seated in a comfortable chair 60 cm away from a computer monitor on which task stimuli were presented, and were instructed to refrain from excessive blinking or moving during data acquisition. Electrophysiological data were collected using two MS windows–compatible computers and a 64‐channel BioSemi ActiveTwo amplifier. The first computer used Presentation software to deliver the stimuli, accept responses, and send digital triggers to the other computer indicating when a stimulus or response occurred. The second computer acquired EEG data using BioSemi software and amplifier (BioSemi B.V., Amsterdam, Netherlands) and all digital triggers. All EEG signals were low‐pass filtered using a fifth‐order sinc filter with a half‐power cutoff of 204.8 Hz then digitized at 1024 Hz during data collection. EEG activity was recorded using sintered Ag‐AgCl active electrodes placed in accordance with the 10–20 International System (Jasper 1958). The participant's nose was used as the reference. Six electrodes were placed on the participant's face to measure electrooculogram. These electrodes were placed above, below, and medial to the canthus of each eye. All offsets were kept below 10 kΩ.

### ERP data reduction

Preprocessing included down sampling to 512 Hz, bad channel detection and replacement, epoching, independent component analysis (ICA)‐based eye‐blink removal, and low‐pass filtered at 15 Hz. Bad channels were identified as having activity 4 SD away from the mean of all other nonocular channels. These channels were replaced using the mean of surrounding electrodes. ERP epochs were defined from 1000 ms pre‐ to 2000 ms poststimulus onset. We investigated stimulus‐locked ERP components elicited by task‐irrelevant distractor stimuli in each task. In the VO‐R task, this was “distractor” stimuli, or repeated low‐probability task‐irrelevant stimuli. In the VO‐D task, this was low‐probability task‐irrelevant novel stimuli. In the Go/NoGo task this was equal probability (as the target stimulus) NoGo stimuli. The epoched data were eye‐blink corrected using an ICA technique. The ICA utility in the EEGLab software (Delorme and Makeig 2004) was used to derive components then, using a template‐matching algorithm (Jung et al. 2000), blink components were identified and removed from the data. Individual subject ICA decompositions where no eye blinks were identified and removed were visually inspected to identify eye‐blink components that, when present, were removed.

Based on previous findings (Bauer 1997; Bauer and Hesselbrock 1999), we extracted the mean amplitude of the N200 and P3a at FCz. The components were specifically defined for each task relative to stimulus onset: VO‐R: the N200 window was defined between 275 and 400 ms. The P3a window was defined 350 and 550 ms; VO‐D: The N200 window was defined between 250 and 400 ms. The P3a window was defined between 350 and 550 ms; Go/NoGo: The N200 window was defined between 180 and 400 ms. The P3a window was defined between 345 and 650 ms. Each component was baseline corrected using a −110 ms prestimulus onset. Within each trial and across all collected scalp electrodes, individual electrode trials for which activity exceeded ±100 *μ*V were omitted from analyses. Applying these criteria, electrode trials for each task were excluded (VO‐R: 20.36%; VO‐D: 18.36%; Go/NoGo: 21.51%).

An additional data reduction method, principal component analysis (PCA; Chapman and McCarry 1995), was also performed separately for each task. This method is optimal for ERP data analysis because classic windowed component time‐domain measures of ERP are inadequate at separating the inherently overlapping ERP components (Dien et al. 2007). We have previously used this method highlighting that PCA measures are more sensitive than windowed components in predicting outcomes (Steele et al. 2014). All scalp electrodes were used in the PCA definition of a five‐component solution was extracted for VO‐R and six‐component solution was extracted for VO‐D and Go/NoGo accounting for 93.02%, 94.55%, and 92.72% of the variance, respectively. Mean measures (i.e., amplitude) in time‐domain windowed components and PCA extracted from FCz are presented below. Effects that did not reach statistical trend (*P *>* *0.10) are not reported.

### Analytical strategy

Response accuracy, ERP time‐domain, and PCA measures were compared between the completion and discontinuation groups using independent sample *t*‐test. Classification using support vector machine (SVM) with two‐nested leave‐one‐out validation (to avoid using the testing data in selecting and training the model; for a detailed mathematical formulation of SVM, see Burges 1998) was conducted to predict treatment completion. Sequential backward feature selection (Jain et al. 2000) was used to identify which covariates were most useful in predicting treatment completion. Feature selection was applied to the covariates to identify the most useful variables in predicting drug treatment completion. Specific implementation of SVM with feature selection is thoroughly described in Steele et al. (2014). These steps were carried out for each task to best identify task‐specific predictions of treatment outcome. Specifically, these tests were designed to identify whether the P3a elicited by a distractor stimuli would predict treatment completion across three tasks that modulated this distractor stimulus (i.e., repetition and frequency of the distractor stimulus).

Five SVM classification models to predict treatment completion were performed for each task. Three simple models containing only the task‐specific covariates identified in the feature selection step, only the time‐domain measures (e.g., mean N2 and P300 from each task), and only the PCA measures identified in the feature selection step. Two additional models combined either the time‐domain or PCA measures with the covariates in a predictive model (Table 2). The best models proved to be the model from each task that only included the PCA measure. Outcome measures from the SVM included: overall accuracy, specificity, sensitivity, positive predictive power, and negative predictive power. Overall accuracy is a measure of how well, overall, the model accurately assigned an individual to the correct group (i.e., a completer assigned to the completer group). Specificity is the measure of how well the model identified who will complete drug treatment, and sensitivity is the measure of how well the model identified who will discontinue drug treatment. Positive predictive value represents the ratio of individuals who discontinued treatment to combined individuals identified correctly and incorrectly to be in the discontinued group. Negative predictive value represents the ratio of individuals who completed treatment to combined individuals identified correctly and incorrectly to be in the completion group.

**Table 2:** Support vector machine models predicting treatment completion

	Covariates (%)	Time‐domain measures (%)	PCA measures (%)	Covariates with TD measures (%)	Covariates with PCA measures (%)
VO‐R					
Overall classification rate	68.97	64.89	67.02	65.52	70.69
Specificity	72.92	66.23	66.23	64.58	75.00
Sensitivity	50.00	58.82	70.59	70.00	50.00
Positive predictive value	27.78	27.78	31.58	29.17	29.41
Negative predictive value	87.50	87.93	91.07	91.18	87.80
VO‐D					
Overall classification rate	62.07	71.88	70.83	58.62	60.34
Specificity	67.35	72.73	70.13	55.10	57.14
Sensitivity	33.33	68.42	73.38	77.78	77.78
Positive predictive value	15.79	38.24	37.84	24.14	25.00
Negative predictive value	84.92	90.32	91.53	93.10	93.33
Go/NoGo					
Overall classification rate	67.50	63.64	68.18	72.50	57.50
Specificity	69.70	64.81	66.67	78.79	51.52
Sensitivity	57.14	58.33	75.00	42.86	85.71
Positive predictive value	28.57	26.92	33.33	30.00	27.27
Negative predictive value	88.46	87.50	92.31	86.67	94.44

Five support vector machine (SVM) models predicting drug treatment completion were computed for each task. In each case, the five models included (1) the covariates identified in feature selection; (2) the time‐domain mean measures; (3) the PCA mean measures identified in feature selection; (4) the covariates identified in feature selection and the time‐domain mean measurements; (5) the covariates and PCA mean measures identified in feature selection.

VO‐R: The covariates identified in feature selection were PCL‐R Factor 1, PCL‐R Factor 2, drug use (total months of abuse), and three measures from the URICA (precontemplation, contemplation, & action). Four of the PCA measures were selected as well (PC1, PC2, PC3, & PC4). VO‐D: The covariates identified in feature selection were IQ, BDI total score, and all four of the measures from the URICA (precontemplation, contemplation, action, & Maintenance). Four of the PCA measures were selected as well (PC1, PC3, PC4, & PC5). Go/NoGo: The covariates identified in feature selection were PCL‐R Factor 1, PCL‐R Factor 2, age, trait anxiety, and three measures from the URICA (contemplation, action, & maintenance). Two of the PCA measures were selected as well (PC1 & PC5). Specificity is the measure of how well the model identified who will complete drug treatment and sensitivity is the measure of how well the model identified who will discontinue drug treatment. Positive predictive value represents the ratio of individuals who discontinued treatment to combined individuals identified correctly and incorrectly to be in the discontinued group. Negative predictive value represents the ratio of individuals who completed treatment to combined individuals identified correctly and incorrectly to be in the completion group.

#### VO‐R task

Of the 12 covariates, six (Psychopathy Checklist‐Revised [PCL‐R] Factor 1 and Factor 2, drug use [total months of abuse], the Pre‐Contemplation, Contemplation and Action scales of the University of Rhode Island Change Assessment [URICA]) were identified to be useful predictors, and were subsequently included in classification analyses. Of the five principal components, four were selected (PC1, PC2, PC3, & PC4).

#### VO‐D task

Of the 12 covariates, six (IQ, BDI total score, the Pre‐Contemplation, Contemplation, Action, and Maintenance scales of the URICA) were identified to be useful predictors and were subsequently included in classification analyses. Of the six principal components, four were selected (PC1, PC3, PC4, & PC5).

#### Go/NoGo task

Of the 12 covariates, nine (PCL‐R Factor 1 and Factor 2, Age, Trait Anxiety, the Contemplation, Action, and Maintenance scales of the University of Rhode Island Change Assessment [URICA]) were identified to be useful predictors, and were subsequently included in classification analyses. Of the six principal components, two were selected (PC1 & PC5).

## Results

### Behavioral results

Response accuracy for each task was analyzed. To test group differences, separate independent samples *t*‐tests were conducted for the novel condition in each task. Groups only differed in accuracy to novel stimuli in the VO‐D task with individuals who discontinued treatment (M = 99.89%, SD = 0.48%) exhibiting higher accuracy than those individuals who completed treatment (M = 99.30%, SD = 1.84%; *t*(94) = 2.51, *P *=* *0.014. *d *= 0.65). Although statistically significant, this difference likely carries little impact considering the very high accuracy rates in each group. In VO‐R and Go/NoGo, groups did not differ in accuracy rates to novel/distractor stimuli, *t's* *<* 1.

### Event‐related potentials

#### VO‐R task

**Figure 1:**
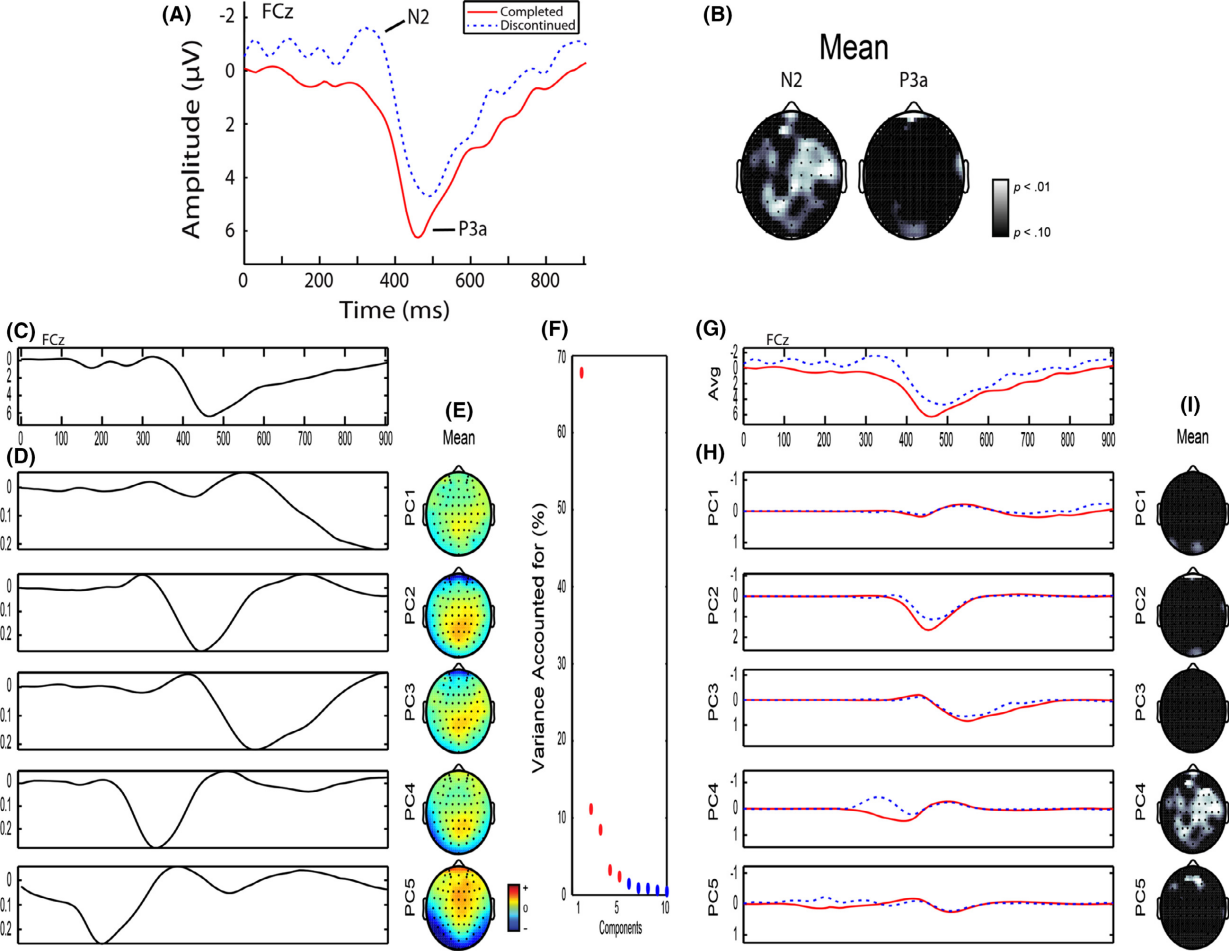
Stimulus‐locked event‐related potential (ERP) and Principal Component Analysis (PCA) of the distractor condition in the VO‐R task: (A) Representative ERP waveform plotted at FCz for each group. Individuals who completed (solid red line) and discontinued (dashed blue line) substance abuse treatment are plotted. ERP components of interest (N2 & P3a) are identified. (B) Topographical statistical difference statistical (black & white) maps are plotted for each component window highlighting individuals who discontinued treatment exhibited reduced N2 amplitude compared to individuals who completed treatment. (C) Grand average waveform plotted at FCz. (D) Principal components extracted accounting for 93.02% of the variance. (E) Topographical depiction of the mean spatial distribution for each principal component. (F) Scree plot of singular values which was used to determine a five‐component solution. (G) Group average waveforms for individuals who completed (solid red line) and discontinued (dashed blue line) substance abuse treatment are plotted at FCz. (H) Principal components plotted by group. (I) Topographical statistical difference (black & white) maps are plotted for each principal component highlighting individuals who discontinued treatment exhibited reduced PC4 amplitude compared to individuals who completed treatment.

Participants in the discontinued group only differed from the completion group in the time‐domain N200 window where they exhibited more negative N200 amplitude, *t*(92) = −2.13, *P *=* *0.036, *d *= 0.47. Only PC4 differed between groups as the discontinued group exhibited more negative PC4 amplitude, *t*(92) = −2.30, *P *=* *0.023, *d *= 0.55 (Fig. 1).

#### VO‐D task

**Figure 2:**
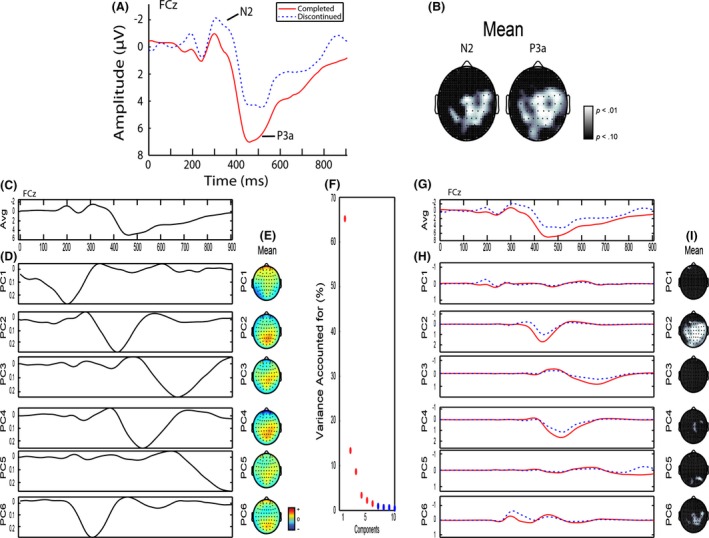
Stimulus‐locked event‐related potential (ERP) and Principal Component Analysis (PCA) of the novel condition in the VO‐D task: (A) Representative ERP waveform plotted at FCz for each group. Individuals who completed (solid red line) and discontinued (dashed blue line) substance abuse treatment are plotted. ERP components of interest (N2 & P3a) are identified. (B) Topographical statistical difference statistical (black & white) maps are plotted for each component window highlighting individuals who discontinued treatment exhibited reduced N2 and P3a amplitude compared to individuals who completed treatment. (C) Grand average waveform plotted at FCz. (D) Principal components extracted accounting for 94.55% of the variance. (E) Topographical depiction of the mean spatial distribution for each principal component. (F) Scree plot of singular values which was used to determine a six‐component solution. (G) Group average waveforms for individuals who completed (solid red line) and discontinued (dashed blue line) substance abuse treatment are plotted at FCz. (H) Principal components plotted by group. (I) Topographical statistical difference (black & white) maps are plotted for each principal component highlighting individuals who discontinued treatment exhibited reduced PC2 amplitude compared to individuals who completed treatment.

Participants in the discontinued group only differed from the completion group in the time‐domain P300 window where they exhibited less positive P300 amplitude, *t*(94) = −1.99, *P *=* *0.049, *d *= 0.41. Only PC5 differed between groups with the discontinued group exhibiting less positive PC5 amplitude, *t*(94) = −2.07, *P *=* *0.041, *d *= 0.44 (Fig. 2).

#### Go/NoGo task

**Figure 3:**
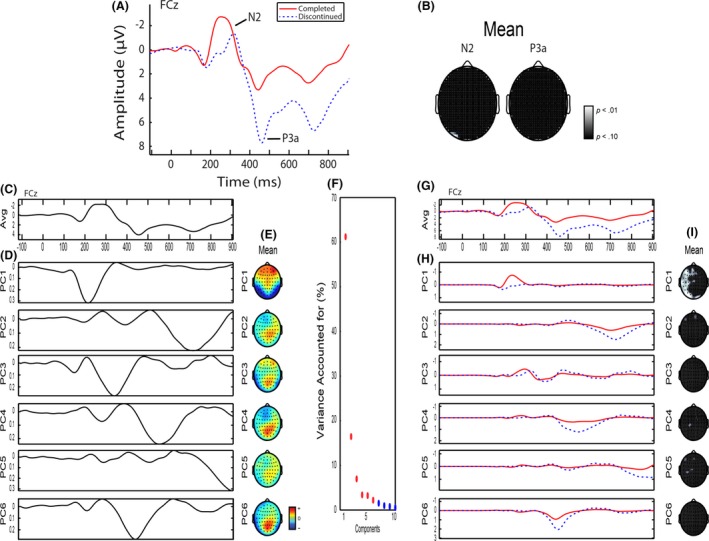
Stimulus‐locked event‐related potential (ERP) and Principal Component Analysis (PCA) of the NoGo condition in the Go/NoGo task: (A) Representative ERP waveform plotted at FCz for each group. Individuals who completed (solid red line) and discontinued (dashed blue line) substance abuse treatment are plotted. ERP components of interest (N2 & P3a) are identified. (B) Topographical statistical difference statistical (black & white) maps are plotted for each component window highlighting individuals who discontinued treatment did not exhibit amplitude differences compared to individuals who completed treatment. (C) Grand average waveform plotted at FCz. (D) Principal components extracted accounting for 92.72% of the variance. (E) Topographical depiction of the mean spatial distribution for each principal component. (F) Scree plot of singular values which was used to determine a six‐component solution. (G) Group average waveforms for individuals who completed (solid red line) and discontinued (dashed blue line) substance abuse treatment are plotted at FCz. (H) Principal components plotted by group. (I) Topographical statistical difference (black & white) maps are plotted for each principal component highlighting individuals who discontinued treatment exhibited left lateral differences from individuals who completed treatment measured in PC1.

No time‐domain differences were found between groups. PC4 and PC5 were marginally different between groups with the discontinued group exhibited more positive amplitude; *t*(64) = 1.80, *P *=* *0.076, *d *= 0.41, *t*(64) = 1.73, *P *=* *0.088, *d *= 0.37, respectively (Fig. 3).

### Classification with support vector machine

#### VO‐R task

Overall 67.02% of participants were correctly identified with the completion group (specificity), 66.23% slightly less well identified than the discontinuation group (sensitivity) 70.59%.

#### VO‐D task

Overall 70.83% of participants were correctly identified with the completion group (specificity), 70.13% slightly less well identified than the discontinuation group (sensitivity) 73.38%.

#### Go/NoGo task

Overall 68.18% of participants were correctly identified with the completion group (specificity), 66.67% less well identified than the discontinuation group (sensitivity) 75.00%. Each of these models provided good group identification without overly biasing group identification to either the discontinuation or completion groups.

## Discussion

This study tested the hypothesis that stimulus‐locked P3a ERP components elicited by distractor stimuli in each of three tasks (VO‐D, VO‐R, Go/NoGo) would discriminate prison inmates who discontinued SUD treatment early from those who received a full therapy dose of at least nine of the 12‐session protocol. Analysis of the behavioral results indicates that inmates who discontinued treatment early showed statistically significant higher accuracy rate to novel stimuli only in the VO‐D task, but this finding is not likely clinically significant due to the high accuracy rate exhibited by both groups. Our predictions regarding ERP components were partially supported, however. Those who discontinued treatment early exhibited a less positive P3a amplitude and a less positive PC5 in the VO‐D task. In the VO‐R task, however, those who discontinued treatment early exhibited a more negative N200 amplitude rather than the hypothesized less positive P3a amplitude. The discontinuation group also displayed less positive PC4 amplitude. For both tasks, the PCA measure was temporally related to the ERP component of interest and should be interpreted as an alternative method of capturing neural correlates related to each ERP component (Dien et al. 2007). Surprisingly, however, there were no time‐domain or principle component differences among the groups in the Go/NoGo task. Support Vector Machine (SVM) models of the three tasks accurately classified individuals who discontinued treatment with the best model accurately classifying 75% of inmates who discontinued treatment (Table 2). Overall, the PCA analyses were more sensitive in differentiating groups than the classic time‐domain windowed approach.

Our pattern of findings are consistent with the context‐updating theory of P300 (Polich 1987). This theory states that after initial sensory processing, an attentional process compares the current stimulus presentation with previous stimuli presentations in working memory (Polich 1987). If no stimulus attribution change is detected, the stimulus context is maintained and only sensory‐evoked potentials are recorded (e.g., N100, P200, N200). If a stimulus attribute change is detected, attentional processes “update” the stimulus representation in working memory and a resulting P300 is recorded (Polich et al. 1985; Fabiani et al. 1986). Our findings suggest that those individuals who discontinued SUD treatment early did not detect the stimulus attribute change in the VO‐R task, where it could be argued that the distractor stimulus is less distinct (white letter “C” on black background, compared to “T” and “X”) than the novel stimuli in the VO‐D task (unique colored geometric figures compared to a white boxes on black backgrounds). Even with these more distinct distractor stimuli in the VO‐D task, participants who discontinued treatment early exhibited less positive P3a suggesting early attentional and working memory deficits among these participants. The lack of significant differences in ERP on the Go/NoGo task between SUD treatment completion groups may suggest that the Go/NoGo task is sufficiently complex to require participants in both groups to devote attentional resources during participation. Task demands have been shown to alter P300 responses related to context‐updating theory (Polich 2007); specifically, the more demanding the cognitive task, the smaller the P300 amplitude (Polich 1987; Kok 2001). The implication of these findings is that the participants who discontinued treatment early may have missed the sometimes subtleties that exist in therapy, the relevance to their own lives, and the benefits of treatment.

### Limitations

The limitations of this study should be considered in evaluating the generalizability of these findings. The first limitation is that the Beck Depression Inventory‐II and the University of Rhode Island Change Assessment only had acceptable reliability which may have affected the ability of these scales to predict inmates who would or would not complete treatment. Another limitation of this study was that fewer participants completed the Go/NoGo task which may have been a result of fatigue given the fixed presentation of tasks, and influenced our findings of no group differences on that task rather than task complexity. Relatedly, we were not able to evaluate the tasks separately for each drug of choice due to low numbers of participants reporting heroin as a drug of choice. Also, more work is needed to better understand the N200 and P3a amplitude cutoff that is most predictive of treatment completion. Although our models provided good specificity and sensitivity and our sample sizes in each task were sufficiently large, our treatment completion and discontinuation groups were not of equal size. Additional reports comprising a larger number of individuals who discontinue SUD treatment early will be needed to better understand the phenomena presented here.

Additionally, after inmates discontinued treatment and withdrew from the study we were no longer able to include them in follow‐up assessments or obtain details as to why they discontinued treatment and study participation (e.g., incompatibility with the treatment). Such information would be useful in developing treatment techniques targeting the most frequent reasons for discontinuation.

### Conclusions and implications for treatment

The findings presented here have significant implications for the treatment of substance abuse and dependence among incarcerated individuals. Here, we add ERP measures of deficiencies in orienting and processing of novel and distractor stimuli to early perceptual and error processing (Steele et al. 2014) that are useful in predicting individuals at greatest risk of discontinuing substance abuse treatment programs. Replication of these effects is necessary with incarcerated and nonincarcerated samples. Research on treatment retention and outcomes in individuals with a history of criminal justice involvement consistently find that more treatment is associated with better outcomes (Evans et al. 2011). These improved outcomes include reduced substance use, improved physical health and psychosocial functioning (McLellan et al. 1992; Landry 1994), and reduced recidivism (Farabee et al. 1998; Harrell and Roman 2001).

The identification of neurocognitive processes that predict treatment discontinuation in prison inmates represents a significant improvement over traditional approaches to predicting treatment continuation, such as client treatment motivation and social desirability (Zemore 2012), which have been notoriously poor predictors. Neurocognitive predictors of treatment completion allow for the development of proxies that correlate with the neurocognitive deficits that place individuals at risk for treatment discontinuation. The proxies may be useful in helping treatment settings identify who may be at highest risk for treatment discontinuation, and may then be able to provide adjunctive treatments to ameliorate this risk. For example, despite its nascence, there is some evidence suggesting that working memory training may improve concurrent cognitive processing (Olesen et al. 2003; Jaeggi et al. 2008; Morrison and Chein 2011). Future studies should investigate working memory training as an adjunct to substance abuse treatment in participants at high risk for treatment discontinuation to determine if such training improves processing of treatment‐related information, and thereby improves treatment retention and outcomes.

## Conflict of Interest

None declared.

## Supporting information


**Table S1.** Descriptive statistics and independent samples *t*‐tests for variables used as covariates – VOR only.
**Table S2.** Descriptive statistics and independent samples *t*‐tests for variables used as covariates – VOD only.
**Table S3.** Descriptive statistics and independent samples *t*‐tests for variables used as covariates – Go10 only.
