# Supplementary material for: Brain potentials predict substance abuse treatment completion in a prison sample
Source: Brain Behav. 2016 May 31;6(8):e00501. doi: 10.1002/brb3.501 (PMC4893048; doi:10.1002/brb3.501)
Supplement: Supplementary file 1 — Table S1. Descriptive statistics and independent samples t‐tests for variables used as covariates – VOR only. Table S2. Descriptive statistics and independent samples t‐tests for variables used as covariates – VOD only. Table S3. Descriptive statistics and independent samples t‐tests for variables used as covariates – Go10 only. [file BRB3-6-e00501-s001.docx]

**Table S1.** Descriptive Statistics and Independent Samples *t*-tests for Variables Used as Covariates- VOR only

All Participants Completed Group Discontinued Group

(*n* = 94 ) (*n* = 77) (*n* = 17)

| Variable | *n* | Mean | SD | *n* | Mean | SD | *n* | Mean | SD | *t* | *df* | *p* |
| --- | --- | --- | --- | --- | --- | --- | --- | --- | --- | --- | --- | --- |
|  |  |  |  |  |  |  |  |  |  |  |  |  |
| Age | 94 | 34.78 | 8.93 | 77 | 34.69 | 8.83 | 17 | 35.18 | 9.67 | 0.20 | 92 | .84 |
| IQ | 93 | 96.43 | 9.97 | 76 | 97.18 | 9.96 | 17 | 93.06 | 9.59 | -1.55 | 91 | .12 |
| Months of Abuse | 84 | 551.67 | 302.08 | 68 | 539.51 | 268.95 | 16 | 603.31 | 422.28 | 0.58 | 18 | .57 |
| PCL-R Total | 79 | 20.09 | 6.14 | 66 | 20.44 | 6.28 | 13 | 18.33 | 5.18 | -1.14 | 77 | .26 |
| PCL-R-F1 | 75 | 5.31 | 2.89 | 62 | 5.58 | 3.01 | 13 | 4.00 | 1.83 | -2.49 | 28 | .02 |
| PCL-R-F2 | 77 | 12.90 | 3.41 | 64 | 13.03 | 3.30 | 13 | 12.23 | 4.00 | -0.77 | 75 | .44 |
| Precontemplation | 89 | 54.89 | 10.47 | 72 | 55.35 | 10.82 | 17 | 52.94 | 8.85 | -0.85 | 87 | .40 |
| Contemplation | 88 | 42.10 | 13.68 | 72 | 41.94 | 13.04 | 16 | 42.81 | 16.73 | 0.23 | 86 | .82 |
| Action | 90 | 48.11 | 12.38 | 73 | 47.47 | 12.53 | 17 | 50.88 | 11.62 | 1.03 | 88 | .31 |
| Maintenance | 89 | 46.91 | 10.16 | 73 | 46.92 | 9.49 | 16 | 46.88 | 13.15 | -0.02 | 87 | .99 |
| State Anxiety | 83 | 40.19 | 13.21 | 67 | 40.57 | 13.32 | 16 | 38.63 | 13.04 | -0.53 | 81 | .60 |
| Trait Anxiety | 81 | 43.77 | 10.25 | 65 | 44.12 | 10.73 | 16 | 42.31 | 8.20 | -0.63 | 79 | .53 |
| Beck’s Depression | 92 | 15.35 | 11.14 | 77 | 15.35 | 11.56 | 15 | 15.33 | 9.01 | -0.01 | 90 | 1.00 |

All participants (*n* = 94) either successfully completed or discontinued a cognitive behavioral substance abuse treatment program. Individuals in the completed group (*n* = 77) include adult incarcerated offenders who successfully completed nine weeks of a cognitive behavioral substance abuse treatment program. Individuals in the discontinued group (*n* = 17) include adult incarcerated offenders who discontinued treatment prior to nine weeks of a cognitive behavioral substance abuse treatment program. Assessments: Intelligence quotient (IQ) was calculated from the Wechsler Adult Intelligence Scale III (WAIS-III; Wechsler, 1997); Months of Abuse is the total number of months of abuse calculated by a modification of the Addiction Severity Index (ASI-X; McLellan et al., 1992); PCL-R-F1 and PCL-R-F2 are the Factor 1 and Factor 2 summary scores from the Psychopathy Checklist – Revised (PCL-R; Hare, 2003); Precontemplation, Contemplation, Action, and Maintenance are summary scores of subscales from the University of Rhode Island Change Assessment (URICA; McConaughy et al., 1983); State Anxiety and Trait Anxiety are summary scores from the State and Trait Anxiety Questions from the State-Trait Anxiety Inventory (STAI; Spielberger et al., 1983); Beck’s Depression is the total score from Beck’s Depression Inventory (BDI-II; Beck et al, 1996).

**Table S2.** Descriptive Statistics and Independent Samples *t*-tests for Variables Used as Covariates- VOD only

All Participants Completed Group Discontinued Group

(*n* = 96) (*n* = 77) (*n* = 19)

| Variable | *n* | Mean | SD | *n* | Mean | SD | *n* | Mean | SD | *t* | *df* | *p* |
| --- | --- | --- | --- | --- | --- | --- | --- | --- | --- | --- | --- | --- |
|  |  |  |  |  |  |  |  |  |  |  |  |  |
| Age | 96 | 34.77 | 8.62 | 77 | 34.99 | 8.81 | 19 | 33.89 | 7.97 | -0.49 | 94 | .62 |
| IQ | 95 | 96.31 | 10.32 | 76 | 96.80 | 10.60 | 19 | 94.32 | 9.12 | -0.94 | 93 | .35 |
| Months of Abuse | 86 | 540.48 | 309.86 | 70 | 534.40 | 298.90 | 16 | 567.06 | 363.57 | 0.38 | 84 | .71 |
| PCL-R Total | 77 | 20.39 | 6.30 | 64 | 20.65 | 6.50 | 13 | 19.10 | 5.23 | -0.81 | 75 | .42 |
| PCL-R-F1 | 74 | 5.46 | 2.99 | 61 | 5.70 | 3.15 | 13 | 4.31 | 1.70 | -2.25 | 32 | .03 |
| PCL-R-F2 | 77 | 13.04 | 3.44 | 64 | 13.13 | 3.35 | 13 | 12.62 | 3.97 | -0.49 | 75 | .63 |
| Precontemplation | 90 | 54.28 | 10.51 | 73 | 54.52 | 10.90 | 17 | 53.24 | 8.83 | -0.45 | 88 | .65 |
| Contemplation | 89 | 41.63 | 13.71 | 72 | 41.88 | 13.33 | 17 | 40.59 | 15.60 | -0.35 | 87 | .73 |
| Action | 90 | 48.61 | 11.92 | 73 | 48.77 | 12.24 | 17 | 47.94 | 10.76 | -0.26 | 88 | .80 |
| Maintenance | 89 | 47.08 | 9.53 | 73 | 46.92 | 9.04 | 16 | 47.81 | 11.83 | 0.34 | 87 | .74 |
| State Anxiety | 80 | 39.35 | 12.58 | 65 | 38.72 | 12.20 | 15 | 42.07 | 14.24 | 0.93 | 78 | .36 |
| Trait Anxiety | 79 | 43.16 | 9.83 | 64 | 42.91 | 9.92 | 15 | 44.27 | 9.70 | 0.48 | 77 | .63 |
| Beck’s Depression | 94 | 15.91 | 11.06 | 77 | 15.61 | 11.09 | 17 | 17.29 | 11.13 | 0.57 | 92 | .57 |

All participants (*n* = 96) either successfully completed or discontinued a cognitive behavioral substance abuse treatment program. Individuals in the completed group (*n* = 77) include adult incarcerated offenders who successfully completed nine weeks of a cognitive behavioral substance abuse treatment program. Individuals in the discontinued group (*n* = 19) include adult incarcerated offenders who discontinued treatment prior to nine weeks of a cognitive behavioral substance abuse treatment program. Assessments: Intelligence quotient (IQ) was calculated from the Wechsler Adult Intelligence Scale III (WAIS-III; Wechsler, 1997); Months of Abuse is the total number of months of abuse calculated by a modification of the Addiction Severity Index (ASI-X; McLellan et al., 1992); PCL-R-F1 and PCL-R-F2 are the Factor 1 and Factor 2 summary scores from the Psychopathy Checklist – Revised (PCL-R; Hare, 2003); Precontemplation, Contemplation, Action, and Maintenance are summary scores of subscales from the University of Rhode Island Change Assessment (URICA; McConaughy et al., 1983); State Anxiety and Trait Anxiety are summary scores from the State and Trait Anxiety Questions from the State-Trait Anxiety Inventory (STAI; Spielberger et al., 1983); Beck’s Depression is the total score from Beck’s Depression Inventory (BDI-II; Beck et al, 1996).

**Table S3.** Descriptive Statistics and Independent Samples *t*-tests for Variables Used as Covariates- Go10 only

All Participants Completed Group Discontinued Group

(*n* = 66) (*n* = 54) (*n* = 12)

| Variable | *n* | Mean | SD | *n* | Mean | SD | *n* | Mean | SD | *t* | *df* | *p* |
| --- | --- | --- | --- | --- | --- | --- | --- | --- | --- | --- | --- | --- |
|  |  |  |  |  |  |  |  |  |  |  |  |  |
| Age | 66 | 34.73 | 9.22 | 54 | 34.70 | 9.35 | 12 | 34.83 | 9.03 | 0.04 | 64 | .97 |
| IQ | 66 | 96.14 | 11.73 | 54 | 96.98 | 11.98 | 12 | 92.33 | 10.08 | -1.25 | 64 | .22 |
| Months of Abuse | 60 | 546.10 | 323.71 | 48 | 539.88 | 320.40 | 12 | 571.00 | 350.10 | 0.30 | 58 | .77 |
| PCL-R Total | 57 | 21.37 | 5.49 | 47 | 21.36 | 5.70 | 10 | 21.43 | 4.62 | 0.04 | 55 | .97 |
| PCL-R-F1 | 55 | 5.67 | 2.89 | 45 | 5.82 | 3.03 | 10 | 5.00 | 2.16 | -0.81 | 53 | .42 |
| PCL-R-F2 | 56 | 13.46 | 3.12 | 46 | 13.28 | 3.18 | 10 | 14.30 | 2.83 | 0.93 | 54 | .36 |
| Precontemplation | 61 | 55.25 | 10.89 | 50 | 54.60 | 10.73 | 11 | 58.18 | 11.68 | 0.99 | 59 | .33 |
| Contemplation | 60 | 42.17 | 13.51 | 49 | 41.63 | 14.38 | 11 | 44.55 | 8.79 | 0.64 | 58 | .52 |
| Action | 61 | 49.59 | 12.46 | 50 | 48.60 | 12.82 | 11 | 54.09 | 9.95 | 1.33 | 59 | .19 |
| Maintenance | 61 | 46.72 | 9.83 | 50 | 46.20 | 9.29 | 11 | 49.09 | 12.21 | 0.88 | 59 | .38 |
| State Anxiety | 54 | 40.04 | 11.63 | 45 | 40.60 | 11.70 | 9 | 37.22 | 11.50 | -0.79 | 52 | .43 |
| Trait Anxiety | 54 | 43.93 | 9.61 | 45 | 44.38 | 9.98 | 9 | 41.67 | 7.52 | -0.77 | 52 | .45 |
| Beck’s Depression | 65 | 15.89 | 11.61 | 54 | 16.22 | 11.77 | 11 | 14.27 | 11.15 | -0.51 | 63 | .62 |

All participants (*n* = 66) either successfully completed or discontinued a cognitive behavioral substance abuse treatment program. Individuals in the completed group (*n* = 54) include adult incarcerated offenders who successfully completed nine weeks of a cognitive behavioral substance abuse treatment program. Individuals in the discontinued group (*n* = 12) include adult incarcerated offenders who discontinued treatment prior to nine weeks of a cognitive behavioral substance abuse treatment program. Assessments: Intelligence quotient (IQ) was calculated from the Wechsler Adult Intelligence Scale III (WAIS-III; Wechsler, 1997); Months of Abuse is the total number of months of abuse calculated by a modification of the Addiction Severity Index (ASI-X; McLellan et al., 1992); PCL-R-F1 and PCL-R-F2 are the Factor 1 and Factor 2 summary scores from the Psychopathy Checklist – Revised (PCL-R; Hare, 2003); Precontemplation, Contemplation, Action, and Maintenance are summary scores of subscales from the University of Rhode Island Change Assessment (URICA; McConaughy et al., 1983); State Anxiety and Trait Anxiety are summary scores from the State and Trait Anxiety Questions from the State-Trait Anxiety Inventory (STAI; Spielberger et al., 1983); Beck’s Depression is the total score from Beck’s Depression Inventory (BDI-II; Beck et al, 1996).
